# *N*-n-Butyl Haloperidol Iodide Ameliorates Cardiomyocytes Hypoxia/Reoxygenation Injury by Extracellular Calcium-Dependent and -Independent Mechanisms

**DOI:** 10.1155/2013/912310

**Published:** 2013-11-12

**Authors:** Yanmei Zhang, Gaoyong Chen, Shuping Zhong, Fuchun Zheng, Fenfei Gao, Yicun Chen, Zhanqin Huang, Wenfeng Cai, Weiqiu Li, Xingping Liu, Yanshan Zheng, Han Xu, Ganggang Shi

**Affiliations:** ^1^Department of Pharmacology, Shantou University Medical College, 22 Xin Ling Road, Shantou, 515041 Guangdong, China; ^2^Department of Biochemistry and Molecular Biology, University of Southern California, Los Angeles, CA 90033, USA; ^3^Department of Pharmacy, The First Affiliated Hospital, Shantou University Medical College, Shantou 515041, Guangdong, China; ^4^Analytical Cytology Laboratory, Shantou University Medical College, Shantou 515041, Guangdong, China; ^5^Department of Cardiovascular Diseases, The First Affiliated Hospital, Shantou University Medical College, Shantou 515041, Guangdong, China

## Abstract

*N*-n-butyl haloperidol iodide (F_2_) has been shown to antagonize myocardial ischemia/reperfusion injury by blocking calcium channels. This study explores the biological functions of ERK pathway in cardiomyocytes hypoxia/reoxygenation injury and clarifies the mechanisms by which F_2_ ameliorates cardiomyocytes hypoxia/reoxygenation injury through the extracellular-calcium-dependent and -independent ERK1/2-related pathways. In extracellularcalcium-containing hypoxia/reoxygenation cardiomyocytes, PKC**α** and ERK1/2 were activated, Egr-1 protein level and cTnI leakage increased, and cell viability decreased. The ERK1/2 inhibitors suppressed extracellular-calcium-containing-hypoxia/reoxygenation-induced Egr-1 overexpression and cardiomyocytes injury. PKC**α** inhibitor downregulated extracellularcalcium-containing-hypoxia/reoxygenation-induced increase in p-ERK1/2 and Egr-1 expression. F_2_ downregulated hypoxia/reoxygenation-induced elevation of p-PKC**α**, p-ERK1/2, and Egr-1 expression and inhibited cardiomyocytes damage. The ERK1/2 and PKC**α** activators antagonized F_2_'s effects. In extracellular-calcium-free-hypoxia/reoxygenation cardiomyocytes, ERK1/2 was activated, LDH and cTnI leakage increased, and cell viability decreased. F_2_ and ERK1/2 inhibitors antagonized extracellular-calcium-free-hypoxia/reoxygenation-induced ERK1/2 activation and suppressed cardiomyocytes damage. The ERK1/2 activator antagonized F_2_'s above effects. F_2_ had no effect on cardiomyocyte cAMP content or PKA and Egr-1 expression. Altogether, ERK activation in extracellular-calcium-containing and extracellular-calcium-free hypoxia/reoxygenation leads to cardiomyocytes damage. F_2_ may ameliorate cardiomyocytes hypoxia/reoxygenation injury by regulating the extracellular-calcium-dependent PKC**α**/ERK1/2/Egr-1 pathway and through the extracellular-calcium-independent ERK1/2 activation independently of the cAMP/PKA pathway or Egr-1 overexpression.

## 1. Introduction

The phenomenon of exacerbated tissue and organ damage produced by the restoration of blood flow after ischemia is known as ischemia/reperfusion (I/R) injury. Studies have demonstrated that this phenomenon takes place in a variety of tissues and organs such as the brain, heart, liver, lungs, kidneys, gastrointestinal tract, limbs, and skin. Myocardial I/R injury is a pathophysiological phenomenon commonly seen after ischemic heart disease and heart surgery. Reducing and eliminating this damage has become a hot topic in the field.

*N*-n-Butyl haloperidol iodide (F_2_) is a new compound synthesized by our group. A series of previous studies have shown that F_2_ has protective effects on *in vivo* myocardial I/R injury and *in vitro* hypoxia/reoxygenation (H/R) injury models [1–4]. Our studies have shown that the F_2_ protection is associated with antagonizing intracellular calcium overload through L-type calcium channels and inhibiting early growth response gene-1 (Egr-1) mRNA and protein overexpression [2, 5–7]. Further analysis has shown that F_2_ is able to inhibit Egr-1 expression through suppression of the H/R-induced classical calcium-dependent PKC*α* translocation/activation. However, it can also activate calcium-independent PKC*ε* translocation/activation to protect cardiomyocytes from sustaining H/R injury [8]. In addition, in cardiac microvascular endothelial cells, which do not have L-type calcium channels, F_2_ still has a protective effect against H/R injury [6, 9–11]. These studies indicate that F_2_ can protect cells from I/R injury through both calcium-dependent and -independent mechanisms. However, it is not clear which signaling pathways are involved. 

The extracellular signal-regulated kinase (ERK1/2) pathway, which has attracted extensive attention in recent years, was the first signal transduction pathway of the MAPK family discovered. It is also the most extensively studied of signal transduction pathway [12]. It is not only involved in the regulation of a variety of cellular physiological functions but also plays an important role in the pathogenesis of a variety of diseases. Numerous studies have shown that the ERK1/2 signaling pathway is closely related to myocardial I/R and H/R injury [13]. Upon I/R or H/R stimulation, ERK1/2 is activated and transducted to the nucleus, phosphorylating serine, and threonine residues of transcription factors and leading to the activation and inactivation of gene transcription and subsequent changes in cell functions [12–14]. Moreover, it was reported that both the Ca^2+^-dependent and -independent pathways are necessary for elevating active ERK to a level sufficient to affect gene expression [15]. To explore the role of ERK1/2 in I/R and H/R injury, we first observed the change of ERK1/2 activity in cardiomyocytes after H/R in the presence and absence of extracellular calcium. Based on these results, we further investigated whether F_2_ protection of cardiomyocytes from H/R injury might take place through its regulation of the calcium-dependent PKC*α*/ERK1/2/Egr-1 signaling pathway. 

Both cAMP and Ca^2+^ are major second messengers. They not only cross-talk by downstream signal molecule but also transduct intracellular signal independently [16]. The cAMP-dependent PKA is the major downstream molecule in the cAMP signaling pathway. The cAMP/PKA activation has been shown to inhibit ERK1/2 activation in Rat-1 cells, NIH/3T3 cells, HEK293 cells, and COS-7 cells [17–19]. In PC12 cells and S49 mouse lymphoma cells, cAMP/PKA acts as an upstream signal to activate ERK1/2 and affect cell function [20, 21]. In cardiomyocytes, the cAMP/PKA signaling pathway is also closely related to ERK1/2. After being activated by isoproterenol, *β*1AR activates the Gs/AC/cAMP/PKA pathways, consequently activates ERK1/2, and causes myocardial apoptosis [22, 23]. These results suggest that the calcium-independent cAMP/PKA/ERK1/2 pathway may be related to H/R-induced myocardial damage. Therefore, in this study, we focused simultaneously on whether the calcium-independent mechanism of F_2_ protection is related to its regulation of the cAMP/PKA/ERK1/2/Egr-1 pathway. 

## 2. Materials and Methods

### 2.1. Culture of Primary Cardiomyocytes

Adult Sprague-Dawley rats (200–250 g) were obtained from Vital River Laboratory Animal Technology Company (Beijing, China). The research protocol was approved by the Medical Animal Care and Welfare Committee of Shantou University Medical College and performed in compliance with the Guide for the Care and Use of Laboratory Animals (NIH Publication, 1996). Primary cardiomyocytes were cultured as described previously with minor modifications [2]. Briefly, neonatal ventricular cardiomyocytes were isolated from 1- to 4-day-old Sprague-Dawley rats with 0.1% trypsin. The dispersed cells were plated in M-199 medium containing 10% fetal bovine serum for 30 min to remove noncardiomyocytes. Then cardiomyocytes, representing 90–95% of total adhering cells, were cultured in the medium with 0.1 mM 5-bromodeoxyuridine for the first 4 days in an incubator with 5% CO_2_ at 37°C. Experiments were performed on day 4 or 5 of the culture.

### 2.2. Preparation of Reagents and Liquid

F_2_ was synthesized in our laboratory. Verapamil was purchased from Shanghai Wellhope Pharmaceuticals (China); ERK inhibitor PD98059 was purchased from Promega (U.S.) and U0126 from Cell Signaling Technology (U.S.); ERK activator EGF was purchased from Pepprotech (U.S.); PKC-*α* inhibitor Gö6976 was purchased from Plymouth Meeting (U.S.); PKC-*α* activator PMA, PKA inhibitor H89, and activator Forskolin were purchased from Sigma (U.S.). Anti-p-PKC*α*, anti-total PKC*α*, anti-PKA, and chemiluminescence luminol reagents were purchased from Santa Cruz Biotechnology (U.S.); anti-p-ERK1/2, anti-total ERK1/2, and anti-Egr-1 were purchased from Cell Signaling Technology (U.S.); anti-*β*-actin and horseradish peroxidase-conjugated secondary antibodies were purchased from Wuhan Boster Biotechnology Limited Company (China); all the other chemicals and reagents were purchased from local agencies. Calcium-containing hypoxia solution was composed of the following: 137 mM NaCl, 12 mM KCl, 0.49 mM MgCl_2_ · 6H_2_O, 0.9 mM of CaCl_2_, 4 mM HEPES, and 20 mM Na lactate. Calcium-free hypoxia solution was composed of the following: 137 mM NaCl, 12 mM KCl, 0.49 mM MgCl_2_ *·*6H_2_O, 1 mM EGTA, 4 mM HEPES, and 20 mM Na lactate. The calcium absent reoxygenation solution was normal medium with 2 mM calcium-chelating EGTA added.

### 2.3. Establishment of Calcium-Containing (Normal Extracellular Calcium) and Calcium-Free (Lacking Extracellular Calcium) H/R Models and Experimental Groups

**Figure 1:**
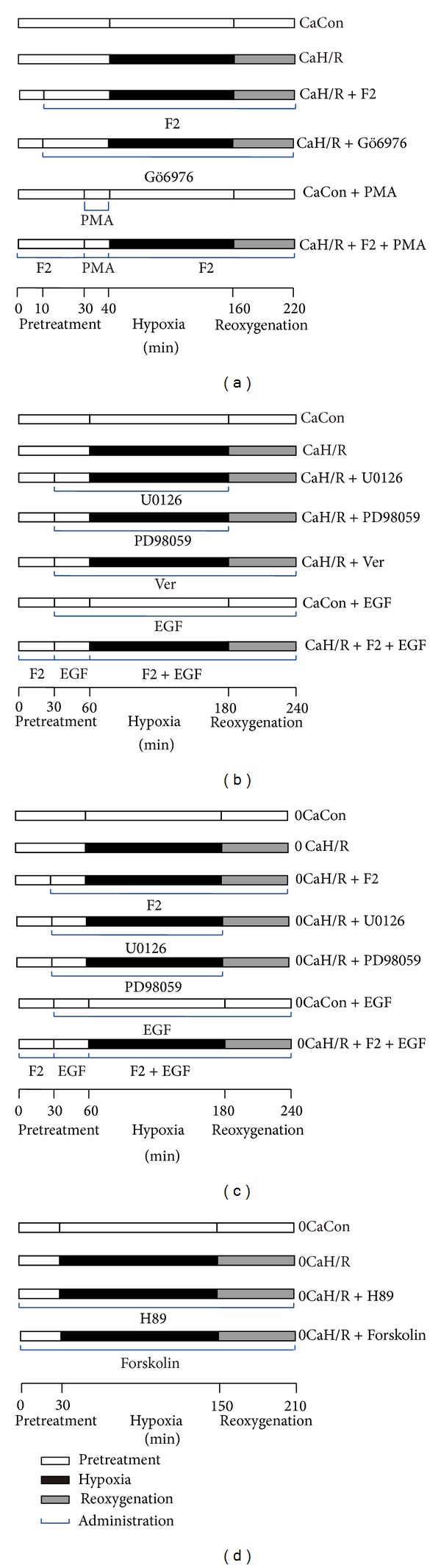
Protocols of experimental grouping and reagent administering. (a) Protocol used to investigate role of ERK1/2 and Egr-1 in extracellular-calcium-containing-H/R injury. (b) Protocol used to investigate role of PKC*α*/ERK1/2/Egr-1 in extracellular-calcium-containing-H/R injury. (c) Protocol used to investigate role of ERK1/2 and Egr-1 in extracellular-calcium-free-H/R injury. (d) Protocol used to investigate role of cAMP/PKA in extracellular-calcium-free-H/R injury. CaCon, calcium-containing normoxia; CaH/R, calcium-containing H/R; 0CaCon, calcium-free normoxic control; 0CaH/R, calcium-free H/R.

Cultured cardiomyocytes were randomly grouped (Figure 1). The calcium-containing-H/R model was established as described previously with 2-hour hypoxia instead of 3-hour hypoxia [2]. F_2_ (1 × 10^−6^ mol/L), Ver (2 × 10^−6^ mol/L), inhibitors (PD98059 (2 × 10^−5^ mol/L), U0126 (2 × 10^−5^ mol/L) and Gö6976 (1 × 10^−6^ mol/L)), and activators (EGF (50 ng/mL) and PMA (1 × 10^−7^ mol/L)) were given in normal medium (for preincubation), hypoxia solution, and/or reoxygenation medium, respectively. The calcium-containing normoxia (CaCon) group was replenished with fresh medium before the experiment and cultured for 3 hours.

The calcium-free-H/R model was established as before only with calcium-free hypoxia solution substituting for calcium-containing hypoxia solution and calcium-free medium for normal medium. F_2_, inhibitors (PD98059, U0126, and H89 (1 × 10^−5^ mol/L)) and activators (EGF and Forskolin (1 × 10^−5^ mol/L)) were also given as above. The calcium-free normoxic control (0CaCon) group was replenished with calcium-free medium before the experiment and cultured for 3 hours. 

### 2.4. Western Blot Analysis

Total protein extracts were prepared from cultured cells using cell lysis buffer containing a protease inhibitor cocktail (aprotinin, leupeptin, pepstatin A, and PMSF). Western blot analysis was performed as described previously with some modifications [2]. The protein concentration was determined using a bicinchoninic acid assay (Bio-Rad, Hercules, CA, USA). Equal amounts of total protein were subjected to SDS-PAGE (10%) followed by electrophoretic transfer to nitrocellulose membranes. The nonspecific binding sites on the membrane were blocked with Tris buffer containing 5% nonfat dry milk for 1 hour. Membranes were probed with anti-p-PKC*α*, anti-total PKC*α*, anti-p-ERK1/2, anti-total ERK1/2, anti-Egr-1, anti-PKA, and anti-*β*-actin antibodies (1 : 5000 dilution for anti-*β*-actin, 1 : 1000 dilution for other antibodies) at 4°C overnight. Blots were then washed three times for 10 min with 20 mM Tris, 150 mM NaCl, and 0.1% Tween 20 (TBST) and incubated with horseradish peroxidase-conjugated secondary antibodies (1 : 5000) for 1 hour. The detection of immunoreactive bands was performed using Western blotting chemiluminescence luminol reagents. The relative densities of protein bands were quantitated using Gel-pro software of densitometric analysis (Media Cybernetics, USA).

### 2.5. Measurements of Cardiac Troponin I (cTnI) and Lactate Dehydrogenase (LDH) Levels in Conditioned Medium

The release of cTnI and LDH was detected in conditioned medium after reoxygenation. The levels of cTnI were measured using an ACS 180 Automated Chemiluminescence System (Bayer Corp., U.S.) with a two-site sandwich immunoassay kit (Bayer Corp., U.S.). The levels of LDH in conditioned medium were determined using test kits (Jiancheng Bioengineering Institute, Nanjing, China):

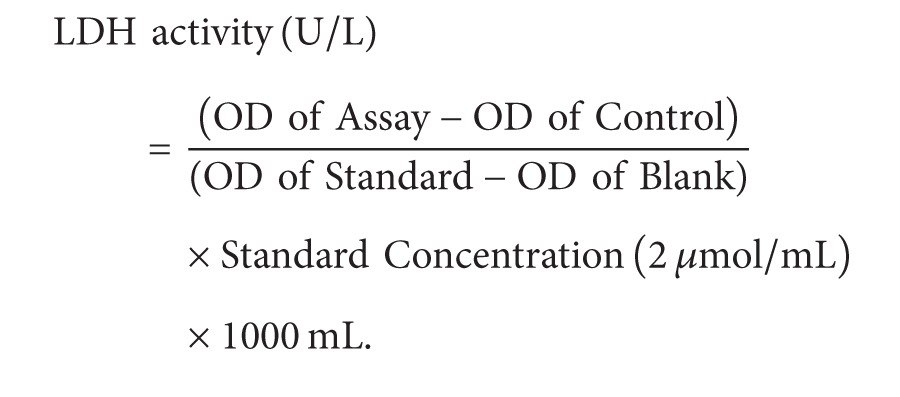
(1)


### 2.6. Assessment of Cardiomyocyte Viability by Cell Counting Kit-8 (CCK-8) Colorimetric Assay

The cardiomyocytes were plated at 5 × 10^4^ cells/well in 96 well plates. Then, 4-5 days later, the cells were treated as described previously. 10 *μ*L of CCK-8 solution was added to 100 *μ*L of reoxygenation solution and the cells were incubated for 1 additional hour after reoxygenation. The absorbance was measured by a microplate reader at 450 nm. 

Consider

(2) Cell  viability(%)=(OD  of  Assay−OD  of  Blank)(OD  of  Control−OD  of  Blank)×100%.

### 2.7. Levels of cAMP in Cultured Cardiomyocytes

The concentration of cAMP in cultured cardiomyocytes was determined by ELISA using a commercially available kit (Enzo Life Sciences International Inc., Switzerland) according to the manufacturer's instructions. All samples and standards were measured in duplicate. Briefly, cardiomyocytes were treated with 0.1 M HCl for 30 minutes and then harvested. After centrifugation, the supernatant was stored at −30°C for later analysis. Fifty microliters of standards and samples were added to a 96-well plate coated with GxR IgG antibody. Then, cAMP-conjugated alkaline phosphatase and cAMP antibody were added to all wells in sequence. After incubation for 2 hours on a plate shaker at 500 rpm, each well was washed three times with wash buffer and then incubated with substrate solution (p-nitrophenyl phosphate) for 1 hour. The reaction was stopped by addition of the stop solution (trisodium phosphate). The plate was read at 405 nm and the concentration of cAMP was calculated according to the standard curve.

### 2.8. Statistical Analysis

Data are shown as the mean ± SEM. The significance of differences was determined using one-way ANOVA followed the by Student-Newman-Keuls test. *P* < 0.05 was considered statistically significant.

## 3. Results

### 3.1. F_2_ Inhibited Calcium-Containing-H/R-Induced ERK1/2 Activation and Consequently Reduced Egr-1 Protein Expression and cTnI Leakage and Improved Cell Viability in Myocardial Cells

#### 3.1.1. Effects of F_2_ on Calcium-Containing-H/R-Induced ERK1/2 Activation and Egr-1 Protein Expression

The ratio of p-ERK1/2 density to total ERK1/2 density reflects the degree of ERK activation. The ratio of total ERK density to *β*-actin density reflects total ERK protein level. The ratio of Egr-1 density to *β*-actin density reflects Egr-1 protein level. In each experiment, the density ratio in the CaCon group was set as 100% and the density ratio in other groups is here expressed relative to CaCon levels.

**Figure 2:**
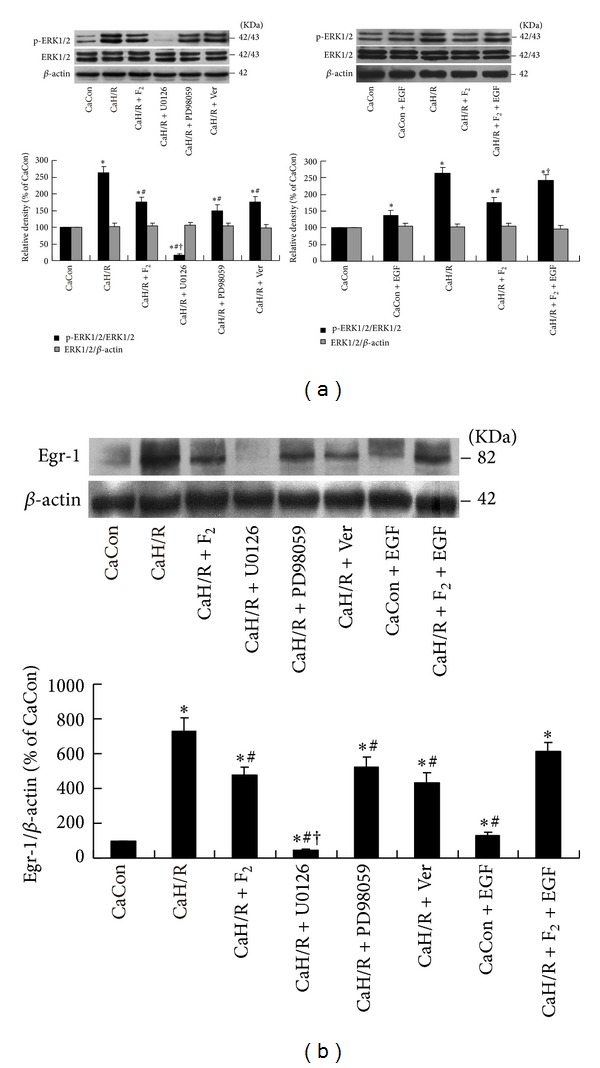
Effects of F_2_, Verapamil, and ERK1/2 inhibitors and activator on p-ERK1/2, total ERK1/2, and Egr-1 expression in extracellular-calcium-containing myocardial H/R by western-blot assay. (a) p-ERK1/2 and total ERK1/2; (b) Egr-1 protein. Quantitative densitometric data were expressed as percentages of the level observed in the CaCon group. All values are expressed as mean ± SEM of at least six individual experiments. *P < 0.05 versus CaCon group; ^#^ *P* < 0.05 versus CaH/R group; ^†^ *P* < 0.05 versus CaH/R+F_2_ group.

As shown in Figure 2, p-ERK1/2 and Egr-1 expression levels were significantly higher in the CaH/R group than in the CaCon group (*P* < 0.05). p-ERK1/2 and Egr-1 expression levels were significantly lower in the CaH/R+F_2_ group, CaH/R+U0126 group, CaH/R+PD98059 group, and CaH/R+Ver group than in the CaH/R group (*P* < 0.05). There was no difference in total ERK1/2 protein expression across different groups (*P* > 0.05). EGF was found to antagonize F_2_ inhibition of H/R-induced upregulation of p-ERK1/2 and Egr-1 expression but had no discernable effect on total ERK1/2 protein expression. EGF activated ERK1/2 under normoxia but did not affect Egr-1 expression. These results suggest that the ERK1/2 signaling pathway mediated calcium-containing-H/R-induced Egr-1 protein upregulation. F_2_ inhibited Egr-1 expression by suppressing the ERK1/2 signaling pathway.

#### 3.1.2. Influence of Inhibition of ERK1/2 Activation on Calcium-Containing-H/R-Induced Leakage of cTnI and Decrease of Cell Viability in Myocardial Cells

cTnI content in cultured cardiomyocyte supernatants was significantly higher and cell viability significantly lower in the CaH/R group than in the CaCon group (*P* < 0.05). F_2_, Verapamil, and ERK1/2 inhibitors U0126 and PD98059 significantly reduced cTnI content and improved cell viability (*P* < 0.05). The ERK1/2 activator EGF was found to antagonize F_2_'s inhibition of cTnI leakage and improvement of cell viability (*P* < 0.05). Under normoxic conditions, EGF had no effect on cTnI content or cell viability (Table 1).

#### 3.1.3. Regulatory Role of F_2_ on Calcium-Containing-H/R-Induced Abnormal PKC*α*/ERK1/2/Egr-1 Pathway

**Figure 3:**
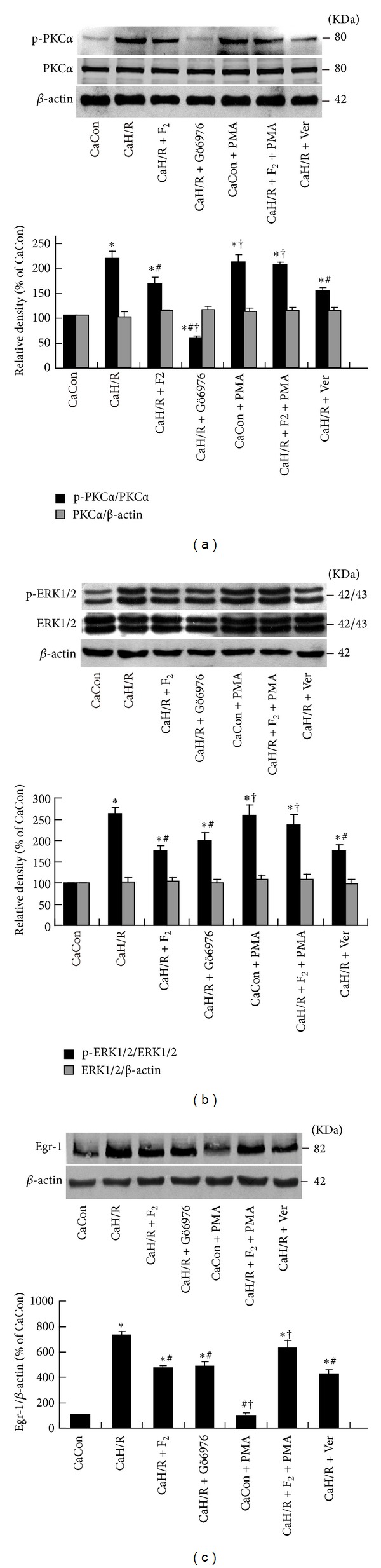
Effects of F_2_, Verapamil, and PKC*α* inhibitor and activator on p-PKC*α*, total PKC*α*, p-ERK1/2, total ERK1/2, and Egr-1 expression in extracellular-calcium-containing myocardial H/R by western-blot assay. (a) p-PKC*α* and total PKC*α* protein levels; (b) p-ERK1/2 and total ERK1/2 protein levels; (c) Egr-1 protein levels. All values are expressed as mean ± S.E.M. of at least six individual experiments. *P < 0.05 versus CaCon group; ^#^ *P* < 0.05 versus CaH/R group; ^†^ *P* < 0.05 versus CaH/R+F_2_ group.

The PKC*α* inhibitor Gö6976 and activator PMA were used to clarify the effects of F_2_ on the PKC*α*/ERK1/2/Egr-1 signaling pathway. The ratio of p-PKC*α* density to total PKC*α* density was used to determine the degree of PKC*α* activation, and the ratio of total PKC*α* density to *β*-actin density was used to determine total PKC*α* protein expression levels. In each experiment, the density ratio in CaCon group was set as 100% and the density ratios in other groups are expressed relative to CaCon group levels (Figure 3).

PKC*α* activity was significantly higher in the CaH/R group than in the CaCon group (*P* < 0.05). PKC*α* activity was significantly lower in the CaH/R + F_2_ group, CaH/R + Gö6976 group, and CaH/R + Ver group than in the CaH/R group (*P* < 0.05). The PKC*α* activator PMA was found to antagonize F_2_ inhibition of calcium-containing-H/R-induced PKC*α* activation in cardiomyocytes (Figure 3(a)). p-ERK1/2 (Figure 3(b)) and Egr-1 (Figure 3(c)) showed the same trend as p-PKC*α*. There were no significant differences in total PKC*α* or total ERK protein expression (*P* > 0.05). Under normoxia, PMA activated PKC*α* and ERK1/2 but did not stimulate Egr-1 protein expression. This study showed that F_2_ inhibited abnormal calcium-containing-H/R-induced activation of the PKC*α*/ERK1/2/Egr-1 signal pathway.

### 3.2. F_2_ Protected Cardiomyocytes from Calcium-Free H/R Injury through Inhibition of ERK1/2 Activation

#### 3.2.1. Effects of F_2_ on the Calcium-Free-H/R-Induced ERK1/2 and Egr-1 Expression and the Relationship of ERK1/2 with Egr-1

**Figure 4:**
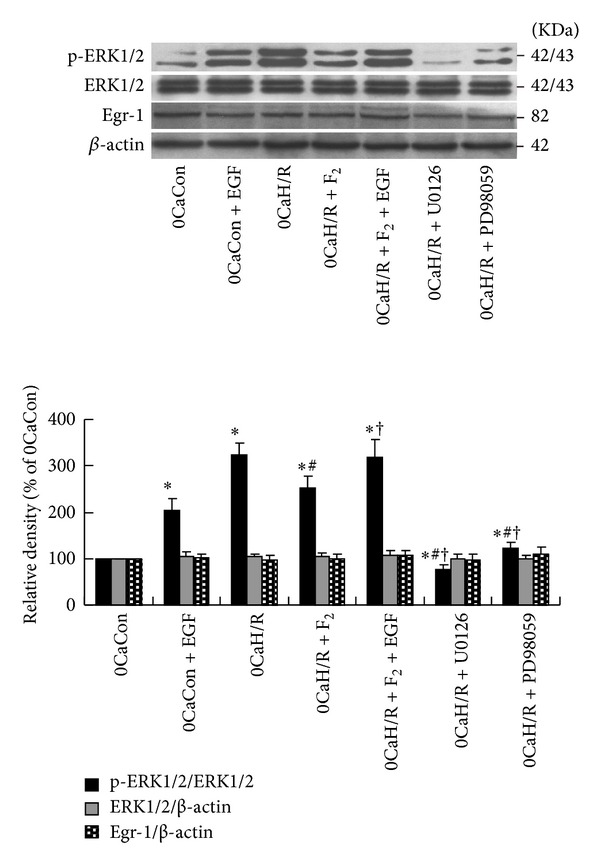
Effects of F_2_, ERK1/2 inhibitors, and activator on p-ERK1/2, total ERK1/2, and Egr-1 expression in extracellular-calcium-free myocardial H/R by western-blot assay. All values are expressed as mean ± S.E.M. of at least six individual experiments. *P < 0.05 versus 0CaCon group; ^#^ *P* < 0.05 versus 0CaH/R group; ^†^ *P* < 0.05 versus 0CaH/R+F_2_ group.

As shown in Figure 4, ERK1/2 activity was significantly higher in the 0CaH/R group than in the 0CaCon group (*P* < 0.05). ERK1/2 activation was significantly lower in the 0CaH/R+F_2_ group, 0CaH/R+U0126 group, and 0CaH/R+PD98059 group than in the 0CaH/R group (*P* < 0.05). ERK1/2 agonist EGF was found to antagonize F_2_ inhibition of calcium-free-H/R-induced p-ERK1/2 upregulation in cardiomyocytes. No difference in total ERK expression was observed between groups (*P* > 0.05). These results suggested that F_2_ could antagonize calcium-free-H/R-induced abnormal activation of ERK1/2 pathway in cardiomyocytes.

No significant changes in Egr-1 protein expression were observed between groups (*P* > 0.05). In the absence of extracellular calcium, H/R was found to activate ERK1/2 but had no effect on Egr-1 protein expression, suggesting that there was no upstream-downstream correlation between ERK1/2 and Egr-1. F_2_ had no effect on Egr-1 protein expression in cardiomyocytes under calcium-free H/R injury conditions.

#### 3.2.2. Effects of Inhibition of ERK1/2 Activation on Calcium-Free-H/R-Induced Leakage of LDH and cTnI and Decrease of Cell Viability

LDH and cTnI levels in cultured cardiomyocyte supernatants were significantly higher, and cell viability was significantly lower in the 0CaH/R group than in the 0CaCon group (*P* < 0.05). F_2_ and ERK1/2 inhibitors U0126 and PD98059 were found to significantly reduce LDH and cTnI concentration and improve cell viability (*P* < 0.05). The ERK1/2 activator EGF was found to antagonize F_2_ inhibition of LDH and cTnI leakage and improvement of cell viability (*P* < 0.05). Under normoxic conditions, EGF was found to have no effect on LDH or cTnI levels or on cell viability (Table 2).

#### 3.2.3. Role of the cAMP/PKA Pathway in F_2_ Protection of Cardiomyocyte from Calcium-Free-H/R-Induced Injury

cAMP/PKA is involved in the regulation of myocardial cell function during I/R by acting as an upstream signaling molecule to activate the ERK1/2 signaling pathway [22]. Like Ca^2+^, cAMP is a transmembrane second messenger. It can be considered a noncalcium second messenger. In this study, we evaluated the effects of F_2_ on cAMP levels and PKA protein expression and examined the effects of PKA inhibitor H89 and activator Forskolin on LDH leakage in cardiomyocytes during calcium-free H/R. The density ratio of PKA to *β*-actin was used to indicate PKA protein expression. The density of PKA in the 0CaCon group was set at 100%, and the density in other groups was calculated relative to these values.

**Figure 5:**
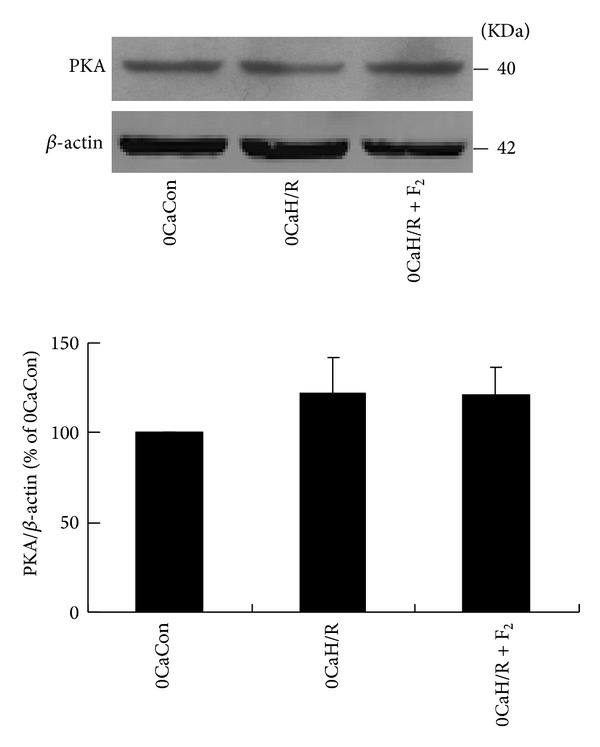
Effects of F_2_ on PKA expression in extracellular-calcium-free myocardial H/R by western-blot assay. All values are expressed as the mean ± S.E.M. of at least six individual experiments.

cAMP levels were lower in the 0CaH/R group than in the 0CaCon group, but the difference was not statistically significant (*P* > 0.05). F_2_ was found to have no effect on cAMP levels in calcium-free H/R. No significant difference in PKA protein was observed between different groups (*P* > 0.05). LDH levels in cultured cell supernatants were significantly higher in the 0CaH/R group than in the 0CaCon group (*P* < 0.05). However, H89 and Forskolin were found to have no effect on LDH levels in cardiomyocytes under calcium-free H/R conditions (Tables 3 and 4 and Figure 5).

## 4. Discussion

### 4.1. Effects of ERK1/2 Activation by H/R Stimulation on Cardiomyocyte Damage

The ERK1/2 pathway is an important cell signaling pathway. It can transfer extracellular information into the nuclei and mediate the ultimate cellular reaction. The studies have shown that the ERK1/2 signaling pathway is involved in I/R injury in a variety of tissues and organs, especially in myocardial tissue [13, 24–27]. In this study, we focused on the relationship between myocardial H/R injury and the ERK1/2 signaling pathway. Our results show that H/R stimulation activated ERK1/2 in both the presence and absence of calcium. This was demonstrated by increased levels of p-ERK1/2 and unchanged levels of total ERK. The ERK1/2-specific inhibitors U0126 and PD98059 effectively inhibited calcium-containing- and calcium-free-H/R-induced ERK1/2 activation, leading to reduced cell damage, as demonstrated by reduced levels of LDH and cTnI leakage, and improved cell viability. These indicate that ERK1/2 activation caused cardiomyocyte damage in both calcium-containing- and calcium-free-H/R stimulation.

Although studies have suggested that activation of the ERK1/2 pathway may promote cell survival [28, 29], our results have shown that the activation of ERK1/2 signaling pathway led to cell damage under myocardial H/R conditions. This result has been supported by many studies, such as the study conducted by Kang et al., which demonstrated that activated ERK1/2 induced by H/R might be novel drug target in cardiomyocytes [30], and the studies of Tsoporis et al. and Liu et al., where they found that the activation of ERK1/2-p53 signaling pathway caused cardiomyocyte apoptosis after myocardial infarction or administration of anticancer drug doxorubicin [31, 32]. In this way, ERK1/2 has a significant impact on the pathophysiological status of cells, but its role may be different in various cell types and experimental models. We believe that the ERK1/2 may play different roles during different stages of I/R: the activation of ERK1/2 in a very short period of I/R may initiate the endogenous protective processes, such as in ischemic preconditioning, but when accompanied by the extension of I/R processes, ERK1/2 activation may initiate injury signals, leading to cell injury [33, 34]. 

### 4.2. Role of Egr-1 Expression Inhibition through the Calcium-Dependent PKC*α*/ERK1/2/Egr-1 Pathways on F_2_ Protection against Myocardial H/R Injury

Our preliminary results showed that blocking L-type calcium channels can inhibit calcium influx and reduce intracellular calcium overload, thereby inhibiting calcium-dependent PKC*α* activation and subsequent abnormal expression of Egr-1. This is one of the important calcium-dependent mechanisms underlying F_2_ protection from H/R-induced myocardial cell injury. However, it is not clear whether ERK mediates signal transduction between PKC*α* and Egr-1.

We found in one previous study that H/R could induce PKC*α* translocation from soluble fraction to particulate fraction in cardiomyocytes [8]. In the present study, we also found that H/R could activate PKC*α* by increasing its phosphorylation. We also observed that the PKC*α* inhibitor Gő6976 inhibited both p-ERK1/2 and Egr-1 protein overexpression, indicating that PKC*α* activation has an important impact on ERK1/2 activation and Egr-1 overexpression, which means that both ERK1/2 and Egr-1 are downstream molecules of PKC*α* signal pathway. Using the ERK1/2 inhibitors U0126 and PD98059, we found ERK1/2 to be an upstream signaling molecule of Egr-1. In the present study, we proved that H/R caused abnormal activation of PKC*α*/ERK1/2/Egr-1 pathway, leading to a series of cellular injuries.

In this study, p-PKC*α*, p-ERK1/2, and Egr-1 protein expression decreased after F_2_ treatment, but total PKC*α* and ERK1/2 protein expression did not change, suggesting that F_2_ can also inhibit PKC*α* and ERK1/2 activation in addition to suppressing Egr-1. The PKC*α* activator PMA can inhibit F_2_ downregulation of p-PKC*α* expression and downregulation of p-ERK1/2 and egr-1 protein expression, suggesting that F_2_ inhibition of ERK1/2 and Egr-1 is dependent on its inhibition of PKC*α* activation. The ERK1/2 activator EGF can inhibit F_2_ downregulation of p-ERK1/2 activation and Egr-1 protein expression and F_2_ protection of cardiomyocytes (including inhibition of cTnI leakage and improvement of cell viability), suggesting that F_2_ protection of cardiomyocytes is dependent on its inhibition of ERK1/2 activation and subsequent downregulation of Egr-1 protein expression. In this way, we proved that F_2_ inhibition of ERK1/2 activation is PKC*α*-dependent and that F_2_ inhibition of Egr-1 overexpression is ERK1/2-dependent, suggesting that F_2_ protection of cardiomyocytes under H/R conditions takes place through its regulation of the abnormal PKC*α*/ERK1/2/Egr-1 signaling pathway. In addition, in this study, we used Verapamil as a positive control for the calcium antagonist and found that Verapamil, like F_2_, has an effect on p-PKC*α*, p-ERK1/2, and Egr-1 protein expression and protects cardiomyocytes from a series of H/R injuries. This suggests that both F_2_ and Verapamil can regulate the abnormal PKC*α*/ERK1/2/Egr-1 pathway, which might be initiated through the regulation of calcium. This hypothesis was supported by the fact that PKC*α* is a calcium-dependent kinase. Under normoxic conditions, Egr-1 expression was low and PMA treatment activated ERK1/2 but did not stimulate Egr-1 protein expression. Similarly, the ERK1/2 activator EGF did not cause Egr-1 protein overexpression or cell damage. These results suggest that the ERK1/2-related cell signaling network is very complicated and the specific intracellular microenvironment at H/R stimulation determines ERK1/2 downstream signaling and its ultimate functions. 

### 4.3. Inhibition of ERK1/2 Activation Is One of the Extracellular Calcium-Independent Mechanisms for F_2_ Protection against Myocardial H/R Injury

Intracellular calcium overload is an important cause of I/R injury. Calcium antagonists can antagonize intracellular calcium overload and protect cardiomyocytes from I/R injury. However, Hempel et al. found that Nifedipine had no effect on ischemia-induced intracellular calcium increases in endothelial cells but it could prevent ischemia-induced PKC translocation and ameliorate increased ischemia-induced endothelial cell permeability [35]. A study performed by Eickelberg et al. showed that Amlodipine, Diltiazem, and Verapamil could regulate transcription factor NF-IL6 and NF-*κ*B in an intracellular-calcium-independent manner [36]. These results indicate that calcium antagonists can affect cell function in ways other than blockage of calcium channels and affecting intracellular calcium levels. Our preliminary results also showed that F_2_, a new L-type calcium antagonist, can not only activate calcium-independent PKC*ε* through translocation in rat cardiomyocytes at the H/R stimulation and protect cardiomyocytes but also protect rat coronary endothelial cells, which do not have L-type calcium channels, from H/R injury [6, 8–11]. We therefore have reason to speculate that F_2_ can protect myocardial cells from H/R injury in an extracellular-calcium-independent manner.

Our results showed that F_2_ inhibited calcium-free-H/R-induced ERK1/2 activation, leading to reduced LDH and cTnI leakage, and improved cell viability. The ERK1/2 activator EGF antagonized F_2_ inhibition of calcium-free-H/R-induced p-ERK1/2 upregulation and inhibited F_2_ protection of cardiomyocytes. These data indicate that in calcium-free H/R, F_2_ can act on ERK1/2 directly or its upstream signal molecule and protect cardiomyocytes from H/R injury. In other words, blocking H/R-induced ERK1/2 pathway activation is an extracellular-calcium-independent mechanism by which F_2_ protects cardiomyocytes. Under normoxic conditions, EGF was found to activate ERK1/2 in the presence or absence of calcium but it did not cause cardiomyocyte injury. However, ERK1/2 activation can cause cell injury in H/R, indicating that although ERK1/2 is a key mediator, it still needs other factors to cause cell injury. 

In the study of ERK1/2-related pathways, our results showed that cAMP levels was lower in the 0CaH/R group than in the 0CaCon group, but this difference were not statistically significant and PKA levels did not change between the groups. In the 0CaH/R group, LDH leakage increased and the PKA activator Forskolin and inhibitor H89 had no effect on calcium-free-H/R-induced LDH leakage, suggesting that calcium-free-H/R-induced cardiomyocyte injury is not mediated by the cAMP/PKA pathway. We also observed that F_2_ had no effect on cAMP concentration or PKA protein expression in calcium-free H/R stimulation, indicating that the extracellular calcium-independent mechanism of F_2_ protection against H/R injury in cardiomyocytes is related to ERK1/2, but the upstream signaling molecule is not related to cAMP/PKA.

This study and our previous studies showed that the myocardial protective effects of F_2_ are related to the inhibition of I/R- and H/R-induced Egr-1 mRNA and protein overexpression. But the present study showed that calcium-free H/R did not cause Egr-1 protein upregulation and that F_2_ had no effect on Egr-1 protein expression in cardiomyocytes in calcium-free H/R. One possible explanation for this is that calcium is involved in the H/R-induced upregulation of Egr-1. We had found that three different types of calcium antagonists, Verapamil, Diltiazem, and Nifedipine, suppressed I/R- and H/R-induced Egr-1 mRNA and protein up-regulation to some extent [7]. Similarly, Lo et al. found that the calcium chelator BAPTA/AM completely inhibited hypoxia-induced Egr-1 overexpression in endothelial cells [37]. We previously observed H/R-induced Egr-1 overexpression and F_2_ protection and inhibition of Egr-1 in microvascular endothelial cells [9–11]. These cells lack L-type calcium channels. However, these specific cells were in an environment with calcium. We therefore speculate that H/R may cause extracellular calcium influx and ultimately Egr-1 overexpression due to countertransportation of calcium by the Na^+^/Ca^2+^ exchanger (NCX) or membrane integrity destruction. F_2_ exhibited its regulation of Egr-1 expression and protective effect on microvascular endothelial cells through the inhibition of NCX outward currents and the subsequent reduction in calcium influx [38]. In this study, EGTA chelated all extracellular calcium and the intracellular-extracellular calcium gradient disappeared. All means of extracellular calcium influx were eliminated and so calcium-dependent Egr-1 overexpression and F_2_'s regulation to it became difficult in calcium-free H/R. 

Although we observed abnormal ERK/Egr-1 pathway activity in calcium-containing H/R model, ERK1/2 inhibitors and activator had no effect on Egr-1 protein expression in calcium-free H/R. This indicates that ERK activation is a master switch to trigger myocardial H/R injury whether in extracellular calcium-containing or calcium-free H/R, nevertheless, its downstream signaling is also determined by the specific intracellular microenvironment such as intracellular calcium concentration. Thus, in the present study, we found that Egr-1 expression did not change with ERK activation when the influx of calcium was eliminated in calcium-free H/R. This result has been supported by Josefsen et al., who found that Egr-1 expression was dependent on Ca^2+^ influx [39]. Of course, other downstream signaling molecules can be involved and worthy of further study.

## 5. Conclusions

In summary, in cultured cardiomyocytes, both extracellular-calcium-containing- and extracellular-calcium-free-H/R were found to activate ERK1/2, leading to cell damage. F_2_ was found to protect cardiomyocytes against H/R injury by regulating extracellular calcium-dependent abnormal PKC*α*/ERK1/2/Egr-1 signaling pathway. F_2_ was also found to protect cardiomyocytes from H/R injury through extracellular calcium-independent mechanisms, which may be related to its suppression of H/R-induced ERK1/2 activation but are not related to the cAMP/PKA signaling pathway or to Egr-1 protein expression.

## Supplementary Material

Our preliminary experiments showed that administration of both the extracellular calcium chelator EGTA and intracellular calcium chelator BAPTA-AM Ca^2+^ could completely chelate calcium, leading to severe cardiomyocyte injury after H/R. We therefore chose to use a sufficient amount of EGTA to completely chelate extracellular Ca^2+^ and establish a extracellular calcium-free H/R ("calcium-free H/R" for short) model to explore the mechanisms of F2 protection on cardiomyocytes. In order to verify whether the preparation is truly calcium-free, we stimulated cardiomyocytes with high levels of potassium in reoxygenation buffer, found no obvious change in intracellular calcium concentration, confirmed that there were no calcium ions in the reoxygenation buffer or calcium levels was no more than intracellular levels, which meets our requirements (Fig. 1).

## Figures and Tables

**Table 1:** Effects of F_2_, Verapamil, and ERK1/2 inhibitors and activator on cTnI level and cell viability in extracellular-calcium-containing myocardial H/R (*n* = 9).

Group	cTnI (ng/mL)	Survival rate (%)
CaCon	0.22 ± 0.01	100.00
CaH/R	0.61 ± 0.03*	47.51 ± 2.15*
CaH/R + F_2_	0.49 ± 0.02^∗#^	64.23 ± 2.78^∗#^
CaH/R + U0126	0.50 ± 0.03^∗#^	63.52 ± 2.46^∗#^
CaH/R + PD98059	0.48 ± 0.02^∗#^	60.40 ± 2.21^∗#^
CaH/R + Ver	0.47 ± 0.02^∗#^	56.85 ± 2.24^∗#^
CaCon + EGF	0.22 ± 0.01^#†^	93.34 ± 3.91^#†^
CaH/R + F_2_ + EGF	0.59 ± 0.01^∗†^	48.54 ± 3.56^∗†^

F_2_, *N*-n-butyl haloperidol iodide; cTnI: cardiac troponin I; H/R: hypoxia/reoxygenation. *P < 0.05 versus CaCon group; ^#^ *P* < 0.05 versus CaH/R group; ^†^ *P* < 0.05 versus CaH/R + F_2_ group.

**Table 2:** Effects of F_2_, and ERK1/2 inhibitors and activator on LDH level, cTnI level, and cell viability in extracellular-calcium-free myocardial H/R (*n* = 9).

Group	LDH (U/mL)	cTnI (ng/mL)	Survival rate (%)
0CaCon	968.65 ± 12.37	0.17 ± 0.01	100.00
0CaH/R	1342.35 ± 15.82*	0.84 ± 0.03*	50.66 ± 1.90*
0CaH/R + F_2_	1135.16 ± 22.33^∗#^	0.62 ± 0.04^∗#^	67.38 ± 2.94^∗#^
0CaH/R + U0126	1155.04 ± 24.24^∗#^	0.69 ± 0.03^∗#^	62.81 ± 4.58^∗#^
0CaH/R + PD98059	1261.39 ± 14.20^∗#^	0.53 ± 0.02^∗#†^	57.73 ± 2.71^∗#^
0CaCon + EGF	1019.90 ± 17.16^#†^	0.17 ± 0.01^#†^	94.01 ± 4.70^#†^
0CaH/R + F_2_ + EGF	1421.43 ± 17.16^∗†^	0.77 ± 0.05^∗†^	53.17 ± 2.48^∗†^

F_2_, *N*-n-butyl haloperidol iodide; LDH: lactate dehydrogenase; cTnI: cardiac troponin I; H/R: hypoxia/reoxygenation. *P < 0.05 versus 0CaCon group; ^#^ *P* < 0.05 versus 0CaH/R group; ^†^ *P* < 0.05 versus 0CaH/R + F_2_ group.

**Table 3:** Effects of F_2_ on cAMP levels of cardiomyocyte in extracellular-calcium-free H/R (*n* = 7).

Group	cAMP (pmol/mL)
0CaCon	2.82 ± 0.55
0CaH/R	1.60 ± 0.35
0CaH/R + F_2_	1.48 ± 0.56

F_2_, *N*-n-butyl haloperidol iodide; cAMP: cyclic adenosine monophosphate; H/R: hypoxia/reoxygenation.

**Table 4:** Effects of PKA inhibitor and activator on LDH leakage of cardiomyocyte in extracellular-calcium-free H/R (*n* = 9).

Group	LDH (U/mL)
0CaCon	952.70 ± 32.02
0CaH/R	1378.33 ± 72.26*
0CaH/R + H89	1389.14 ± 71.65*
0CaH/R + Forskolin	1338.78 ± 51.68*

LDH: lactate dehydrogenase; PKA: protein kinase A; H/R: hypoxia/reoxygenation.*P < 0.05 versus 0CaCon group.

## References

[1] Gao F-F, Shi G-G, Zheng J-H, Liu B (2004). Protective effects on N-n-butyl haloperidol iodide on myocardial ischemia-reperfusion injury in rabbits. *Chinese Journal of Physiology*.

[2] Zhang Y, Shi G, Zheng J (2007). The protective effects of N-n-butyl haloperidol iodide on myocardial ischemia-reperfusion injury in rats by inhibiting Egr-1 overexpression. *Cellular Physiology and Biochemistry*.

[3] Zhang Y-M, Shi G-G, Tang Z (2006). Effects of N-n-butyl haloperidol iodide on myocardial ischemia/reperfusion injury and Egr-1 expression in rat. *Acta Biochimica et Biophysica Sinica*.

[4] Huang Z-Q, Shi G-G, Zheng J-H, Liu B (2003). Effects of N-n-butyl haloperidol iodide on rat myocardial ischemia and reperfusion injury and L-type calcium current. *Acta Pharmacologica Sinica*.

[5] Zhang Y, Shi G, Zheng J (2008). The protective effect of Egr-1 antisense oligodeoxyribonucleotide on myocardial injury induced by ischemia-reperfusion and hypoxia-reoxygenation. *Cellular Physiology and Biochemistry*.

[6] Zhou Y, Zhang Y, Gao F (2010). N-n-butyl haloperidol iodide protects cardiac microvascular endothelial cells from hypoxia/reoxygenation injury by down-regulating egr-1 expression. *Cellular Physiology and Biochemistry*.

[7] Huang Z, Li H, Guo F (2009). Egr-1, the potential target of calcium channel blockers in cardioprotection with ischemia/reperfusion injury in rats. *Cellular Physiology and Biochemistry*.

[8] Wang J-Z, Cai C-Y, Zhang Y-M (2010). N-n-Butyl haloperidol iodide protects against hypoxia/reoxygenation-induced cardiomyocyte injury by modulating protein kinase C activity. *Biochemical Pharmacology*.

[9] Matsui T, Yamagishi S-I, Nakamura K, Inoue H (2007). Bay w 9798, a dihydropyridine structurally related to nifedipine with no calcium channel-blocking properties, inhibits tumour necrosis factor-*α*-induced vascular cell adhesion molecule-1 expression in endothelial cells by suppressing reactive oxygen species generation. *Journal of International Medical Research*.

[10] Yamagishi S-I, Nakamura K, Matsui T (2008). Role of oxidative stress in the development of vascular injury and its therapeutic intervention by nifedipine. *Current Medicinal Chemistry*.

[11] Berkels R, Taubert D, Rosenkranz A, Rösen R (2003). Vascular protective effects of dihydropyridine calcium antagonists. Involvement of endothelial nitric oxide. *Pharmacology*.

[12] Clerk A, Sugden PH (2004). Signaling through the extracellular signal-regulated kinase 1/2 cascade in cardiac myocytes. *Biochemistry and Cell Biology*.

[13] Li D-Y, Tao L, Liu H, Christopher TA, Lopez BL, Ma XL (2006). Role of ERK1/2 in the anti-apoptotic and cardioprotective effects of nitric oxide after myocardial ischemia and reperfusion. *Apoptosis*.

[14] Murphy LO, Blenis J (2006). MAPK signal specificity: the right place at the right time. *Trends in Biochemical Sciences*.

[15] Mao L, Yang L, Tang Q, Samdani S, Zhang G, Wang JQ (2005). The scaffold protein Homer1b/c links metabotropic glutamate receptor 5 to extracellular signal-regulated protein kinase cascades in neurons. *Journal of Neuroscience*.

[16] Andersson DC, Fauconnier J, Yamada T (2011). Mitochondrial production of reactive oxygen species contributes to the *β*-adrenergic stimulation of mouse cardiomycytes. *Journal of Physiology*.

[17] Cook SJ, McCormick F (1993). Inhibition by cAMP of Ras-dependent activation of Raf. *Science*.

[18] Crespo P, Cachero TG, Xu N, Gutkind JS (1995). Dual effect of *β*-adrenergic receptors on mitogen-activated protein kinase. Evidence for a *βγ*-dependent activation and a G*α*(s)-cAMP-mediated inhibition. *Journal of Biological Chemistry*.

[19] Schmitt JM, Stork PJS (2001). Cyclic AMP-mediated inhibition of cell growth requires the small G protein Rap1. *Molecular and Cellular Biology*.

[20] Wan Y, Huang X-Y (1998). Analysis of the G(s)/mitogen-activated protein kinase pathway in mutant S49 cells. *Journal of Biological Chemistry*.

[21] MacNicol MC, MacNicol AM (1999). Nerve growth factor-stimulated B-Raf catalytic activity is refractory to inhibition by cAMP-dependent protein kinase. *Journal of Biological Chemistry*.

[22] Zheng J, Shen H, Xiong Y, Yang X, He J (2010). The *β*1-adrenergic receptor mediates extracellular signal-regulated kinase activation via G*α*s. *Amino Acids*.

[23] Zhu W-Z, Zheng M, Koch WJ, Lefkowitz RJ, Kobilka BK, Xiao R-P (2001). Dual modulation of cell survival and cell death by *β*2-adrenergic signaling in adult mouse cardiac myocytes. *Proceedings of the National Academy of Sciences of the United States of America*.

[24] Xing W-J, Kong F-J, Li G-W (2011). Calcium-sensing receptors induce apoptosis during simulated ischaemia-reperfusion in Buffalo rat liver cells. *Clinical and Experimental Pharmacology and Physiology*.

[25] Yamamoto S, Yamane M, Yoshida O (2011). Activations of mitogen-activated protein kinases and regulation of their downstream molecules after rat lung transplantation from donors after cardiac death. *Transplantation Proceedings*.

[26] Tian H-P, Huang B-S, Zhao J, Hu X-H, Guo J, Li L-X (2009). Non-receptor tyrosine kinase Src is required for ischemia-stimulated neuronal cell proliferation via Raf/ERK/CREB activation in the dentate gyrus. *BMC Neuroscience*.

[27] Alderliesten M, De Graauw M, Oldenampsen J (2007). Extracellular signal-regulated kinase activation during renal ischemia/reperfusion mediates focal adhesion dissolution and renal injury. *American Journal of Pathology*.

[28] Milano G, Von Segesser LK, Morel S (2010). Phosphorylation of phosphatidylinositol-3-kinase-protein kinase B and extracellular signal-regulated kinases 1/2 mediate reoxygenation-induced cardioprotection during hypoxia. *Experimental Biology and Medicine*.

[29] Bueno OF, Molkentin JD (2002). Involvement of extracellular signal-regulated kinases 1/2 in cardiac hypertrophy and cell death. *Circulation Research*.

[30] Kang S-M, Lim S, Song H (2006). Allopurinol modulates reactive oxygen species generation and Ca^2+^ overload in ischemia-reperfused heart and hypoxia-reoxygenated cardiomyocytes. *European Journal of Pharmacology*.

[31] Tsoporis JN, Izhar S, Leong-Poi H, Desjardins J-F, Huttunen HJ, Parker TG (2010). S100B interaction with the receptor for advanced glycation end products (RAGE): a novel receptor-mediated mechanism for myocyte apoptosis postinfarction. *Circulation Research*.

[32] Liu J, Mao W, Ding B, Liang C-S (2008). ERKs/p53 signal transduction pathway is involved in doxorubicin-induced apoptosis in H9c2 cells and cardiomyocytes. *American Journal of Physiology*.

[33] Yang X, Cohen MV, Downey JM (2010). Mechanism of cardioprotection by early ischemic preconditioning. *Cardiovascular Drugs and Therapy*.

[34] Juan-Zhang J-Z, Bian H-J, Li X-X (2010). ERK-MAPK signaling opposes rho-kinase to reduce cardiomyocyte apoptosis in heart ischemic preconditioning. *Molecular Medicine*.

[35] Hempel A, Lindschau C, Maasch C (1999). Calcium antagonists ameliorate ischemia-induced endothelial cell permeability by inhibiting protein kinase C. *Circulation*.

[36] Eickelberg O, Roth M, Mussmann R (1999). Calcium channel blockers activate the interleukin-6 gene via the transcription factors NF-IL6 and NF-*κ*B in primary human vascular smooth muscle cells. *Circulation*.

[37] Lo L-W, Cheng J-J, Chiu J-J, Wung B-S, Liu Y-C, Wang DL (2001). Endothelial exposure to hypoxia induces Egr-1 expression involving PKC*α*-mediated Ras/Raf-1/ERK1/2 pathway. *Journal of Cellular Physiology*.

[38] Huang Y, Gao F, Zhang Y (2012). N-n-Butyl haloperidol iodide inhibits the augmented Na^+^/Ca^2+^ exchanger currents and L-type Ca^2+^ current induced by hypoxia/reoxygenation or H_2_O_2_ in cardiomyocytes. *Biochemical and Biophysical Research Communications*.

[39] Josefsen K, Sørensen LR, Buschard K, Birkenbach M (1999). Glucose induces early growth response gene (Egr-1) expression in pancreatic beta cells. *Diabetologia*.

