# Supplementary material for: N-n-Butyl Haloperidol Iodide Ameliorates Cardiomyocytes Hypoxia/Reoxygenation Injury by Extracellular Calcium-Dependent and -Independent Mechanisms
Source: Oxid Med Cell Longev. 2013 Nov 12;2013:912310. doi: 10.1155/2013/912310 (PMC3857550; doi:10.1155/2013/912310)
Supplement: Supplementary file 1 — Our preliminary experiments showed that administration of both the extracellular calcium chelator EGTA and intracellular calcium chelator BAPTA-AM Ca2+ could completely chelate calcium, leading to severe cardiomyocyte injury after H/R. We therefore chose to use a sufficient amount of EGTA to completely chelate extracellular Ca2+ and establish a extracellular calcium-free H/R ("calcium-free H/R" for short) model to explore the mechanisms of F2 protection on cardiomyocytes. In order to verify whether the preparation is truly calcium-free, we stimulated cardiomyocytes with high levels of potassium in reoxygenation buffer, found no obvious change in intracellular calcium concentration, confirmed that there were no calcium ions in the reoxygenation buffer or calcium levels was no more than intracellular levels, which meets our requirements (Fig. 1). [file 912310.f1.docx]

**
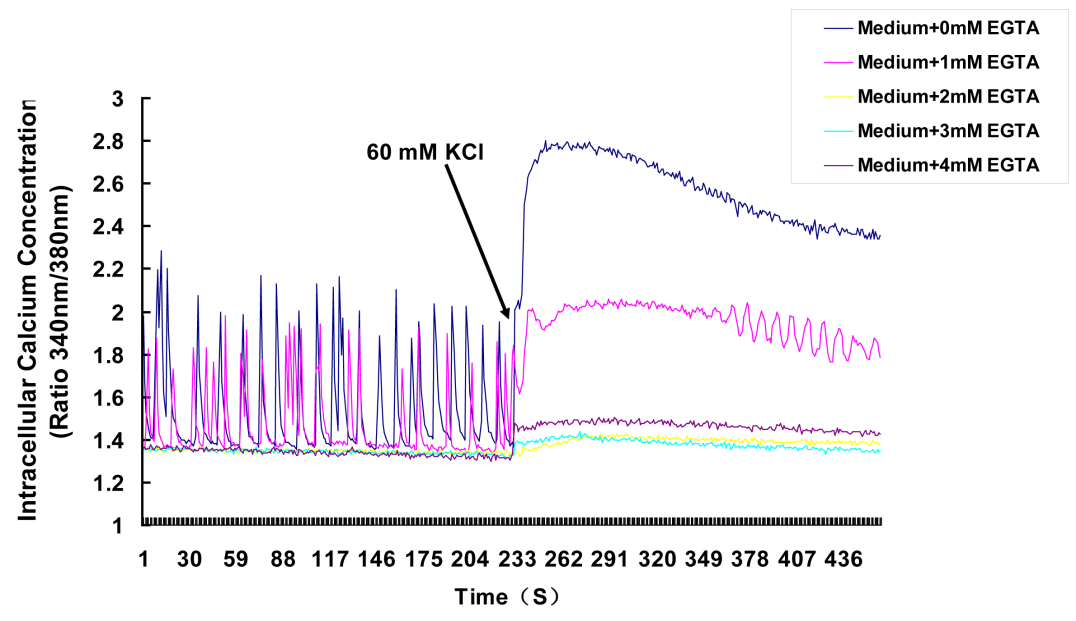
**

Fig. 1 Changes of intracellular calcium concentration after high levels of potassium by a high speed calcium imging system. The intracellular calcium concentration of cardiomyocyte didn’t elevated markedly in the medium pretreated with 2 or more than 2 mM EGTA.
